# Autoimmune Cytopenias in Common Variable Immunodeficiency Are a Diagnostic and Therapeutic Conundrum: An Update

**DOI:** 10.3389/fimmu.2022.869466

**Published:** 2022-06-20

**Authors:** Sanchi Chawla, Prabal Barman, Rahul Tyagi, Ankur Kumar Jindal, Saniya Sharma, Amit Rawat, Surjit Singh

**Affiliations:** Allergy Immunology Unit, Department of Pediatrics, Advanced Pediatrics Centre, Post Graduate Institute of Medical Education and Research, Chandigarh, India

**Keywords:** common variable immunodeficiency (CVID), autoimmune cytopenia (AIC), B cells, lipopolysaccharide (LPS)-responsive beige-like anchor protein (LRBA), cytotoxic T lymphocyte antigen 4 (CLTA-4), B cell activating factor (BAFF), inducible T cell co-stimulator (ICOS)

## Abstract

Common variable immunodeficiency (CVID) is the most common symptomatic primary immunodeficiency (PID). CVID is a heterogenous condition and clinical manifestations may vary from increased susceptibility to infections to autoimmune manifestations, granulomatous disease, polyclonal lymphoproliferation, and increased risk of malignancy. Autoimmune manifestations may, at times, be the first and only clinical presentation of CVID, resulting in diagnostic dilemma for the treating physician.

Autoimmune cytopenias (autoimmune haemolytic anaemia and/or thrombocytopenia) are the most common autoimmune complications seen in patients with CVID. Laboratory investigations such as antinuclear antibodies, direct Coomb’s test and anti-platelet antibodies may not be useful in patients with CVID because of lack of specific antibody response. Moreover, presence of autoimmune cytopenias may pose a significant therapeutic challenge as use of immunosuppressive agents can be contentious in these circumstances. It has been suggested that serum immunoglobulins must be checked in all patients presenting with autoimmune cytopenia such as immune thrombocytopenia or autoimmune haemolytic anaemia.

It has been observed that patients with CVID and autoimmune cytopenias have a different clinical and immunological profile as compared to patients with CVID who do not have an autoimmune footprint. Monogenic defects have been identified in 10-50% of all patients with CVID depending upon the population studied. Monogenic defects are more likely to be identified in patients with CVID with autoimmune complications. Common genetic defects that may lead to CVID with an autoimmune phenotype include *nuclear factor kappa B subunit 1 (NF-kB1), Lipopolysaccharide (LPS)-responsive beige-like anchor protein (LRBA), cytotoxic T lymphocyte antigen 4 (CTLA4), Phosphoinositide 3-kinase (PI3K), inducible T-cell costimulatory (ICOS), IKAROS* and *interferon regulatory factor-2 binding protein 2 (IRF2BP2).*

In this review, we update on recent advances in pathophysiology and management of CVID with autoimmune cytopenias.

## Introduction

Common variable immunodeficiency (CVID) is the most common symptomatic primary immunodeficiency (1–3). CVID is a predominant antibody deficiency disease and there is marked reduction of serum immunoglobulin (IgG) and immunoglobulin (IgA) and/or immunoglobulin (IgM) along with impaired or poor response to vaccines (2). Since the first description of this entity in 1954 (4), there has been a remarkable progress in understanding the clinical phenotype of this disease. CVID is a heterogenous condition and clinical manifestations may vary from increased susceptibility to infections to autoimmune manifestations, granulomatous disease, polyclonal lymphoproliferation, and increased risk of malignancy. Autoimmune manifestations may be seen in 25 to 30% of all patients with CVID and may, at times, be the first and only clinical presentation (2). Such presentations of CVID can result in diagnostic dilemma for the treating physician.

Of the various autoimmune complications seen in patients with CVID, autoimmune cytopenias (autoimmune haemolytic anaemia, thrombocytopenia, Evan’s syndrome, neutropenia and pernicious anaemia) are the most common (5). Laboratory investigations such as antinuclear antibodies, direct Coomb’s test and anti-platelet antibodies may be negative in patients with CVID because of lack of specific antibody responses. Moreover, presence of autoimmune cytopenias may pose a significant therapeutic challenge as use of immunosuppressive agents can be contentious in these circumstances.

It has been observed that patients with CVID and autoimmune cytopenias have a different clinical and immunological profile as compared to patients with CVID who do not have an autoimmune footprint (5). Monogenic defects have also been identified in 10-50% of all patients with CVID depending upon the population studied (6). Monogenic defects are more likely to be identified in patients with CVID with autoimmune complications (7).

This review will elaborate on recent developments in pathophysiology and management of CVID in the context of autoimmune cytopenia.

## Clinical Phenotype of Autoimmune Cytopenia in CVID

Of the various autoimmune complications in CVID, cytopenia has been reported to be the most common complication (5). Recent data from United States Immunodeficiency Network (USIDNET) registry showed that patients with CVID with autoimmune cytopenia had one or more of disease associated non-infectious complications such as lymphoproliferation, liver disease, interstitial lung disease, granulomatous inflammation, enteropathy, other-organ specific autoimmunity and increased risk of lymphoma (8). This complex interplay between autoimmune manifestations in various systems remains an enigma and exact etiopathogenesis remains speculative. In one of the largest cohorts of CVID patients, Gathmann et al. reported a strong association between autoimmunity and enteropathy (9). Another study by Mormille et al. reported splenomegaly in almost all patients with cytopenia (88%) (10). Most studies over last two decades have shown an association of autoimmune cytopenia with splenomegaly and granulomatous disease (**Table 1**).

**Table 1:** Review of studies that have reported the clinical phenotype of autoimmune cytopenia in CVID.

Author, year, country	Number of patients[x/y]^#^	Sex ratio(M: F)	Age at diagnosis (years)	Salient findings	Management
Hermaszewsky et al., 1993, UK (11)	40/240	NA	Biphasic(1-5; 16-20)	12 CVID patients had AIHA, 6 had ITP, 4 had pernicious anaemia and 18 had neutropeniaThrombocytopenia was mild and nearly half of these patients had splenomegalyNeutropenic patients had poor prognosis because of increased infections	Splenectomy was performed in 5 and 2 patients with AIHA and ITP respectively
Cunningham-Rundles et al., 1999, USA (12)	32/248	51:73(15:17)	29 (Male)33 (Female)	Females had a higher predisposition for autoimmunity including cytopenia15 patients had ITP, 12 had AIHA, 3 had pernicious anaemia, 2 had autoimmune neutropenia, 5 had Evan’s syndrome	IVIg and short course steroids
Kainulainen et a.l, 2001, Finland (13)	10/95	52:43	33	Eighteen (19%) patients with CVID had autoimmune manifestations; pernicious anaemia was the commonest (6%) followed by ITP (3%) and AIHA (1%)	NA
Kokron et al., 2004, Brazil (14)	3/71	38:33	15-78	2 patients had haemolytic anaemia, while 1 had pernicious anaemia; 1 female patient had both haemolytic anaemia and Sjoügren Syndrome and 1 male patient had atrophic gastritis and pernicious anaemia	IVIg
Michel et al., 2004, France (15)	21/21	4:3	27 (10-74)	The median age at AITP diagnosis was earlier than the diagnosis of CVIDCVID was diagnosed before the onset of AITP in only 4 patients (19%). It was diagnosed more than 6 months after AITP in 13 cases (62%), and the 2 conditions were diagnosed concomitantly in 4 cases11 patients (52%) had at least 1 autoimmune manifestation other than AITP, among which AIHA (7 cases) and autoimmune neutropenia (5 cases) were more common	The commonest treatment included steroids and IVIg (1-2g/kg). 6 patients needed additional therapy including azathioprine, vincristine and cyclophosphamide4 patients underwent splenectomy for AITP (2 had complete remission and 2 failed to respond). Two patients underwent splenectomy for Evans syndrome
Wang et al., 2005, USA (16)	35/326	16:19	5-66	19 (54%) patients had the 1^st^ episode of thrombocytopenia or haemolytic anaemia prior to the diagnosis of CVID, 11 (32%) were diagnosed concurrently, and 5 (14%) developed one or both of these autoimmune diseases following the diagnosis of CVID; 8 patients with cytopenia also had granulomas	Treatment included corticosteroids, anti-Rh immunoglobulin, and intravenous immunoglobulinEleven patients underwent splenectomy
Carbone et al., 2006, Spain (17)	3/14	4:3	37.4(21-68)	2 patients had ITP and 1 had AIHA	NA
Alachkar et al., 2006, UK (18)	NA/34	25:9	25 (8-51)	Reduced switched memory B cells was associated with a significantly higher prevalence of bronchiectasis, splenomegaly and autoimmunity	NA
Quinti et al., 2007, Italy (19)	97/224*	48:49	26.6 (2-73)	At the time of diagnosis of CVID, autoimmune diseases were the only features in 2.3% of patients while in 11.1% autoimmune diseases were associated with recurrent infections	Steroids and splenectomy (more details NA)
Chapel et al., 2008, UK, Sweden, Germany, France, Czech Republic (20)	40/334	1.4:1	33	There was a statistically significant correlation of splenomegaly with cytopenias, hepatomegaly, and granulomata, but not with solid organ–specific autoimmunity	NA
Wehr et al., 2008, UK, Germany, France, Spain, Netherlands and Czech Republic (21)	43/303	133:169	35 (3-74)	The age of onset of immunodeficiency was delayed in CVID patients with autoimmune manifestations although it was not statistically significant because of low numbers; majority had ITP (64%), followed by AIHA (25%), and 11% had Evan’s syndrome; nine patients had pernicious anaemia; There was no difference between genders; autoimmune cytopenia had significant associations with splenomegaly and granulomatous disease	NA
Ardeniz et al., 2009, Turkey/USA (22)	19/37	13:24	26 (2-59)	7 patients with autoimmune cytopenia also had granulomas (lung and liver) as the predominant manifestation	Steroids used most commonly; 2 patients received cyclosporin, 1 infliximab and 1 rituximab
Mouillot et al., 2010, France (23)	55/313	0.9:1	45 (33-56)	Correlation was noted between decreased switched memory B cells, decrease in naive CD4+ T cells and increase in CD4+CD95+ cells with lymphoproliferation, autoimmune cytopenia, or chronic enteropathyIn addition, lymphoproliferation and cytopenia patients had increase in CD21low B cells and CD4+HLA-DR+ T cells and decreased regulatory T cell	NA
Boileau et., 2011, France (24)	55/311	29:26	29 (16-46)	41 patients (74%) had ITP, 17 patients (31%) had AIHA and 10 patients (18%) had neutropenia. 36 patients in this group developed splenomegaly (65%) and 8 patients developed a granulomatous disease (14%); a significant correlation was found between an increased proportion of CD21low B cells and CVID associated autoimmune cytopenia; in CVID associated autoimmune cytopenia, T cells display an activated phenotype with an increase of HLA-DR and CD95 expression and a decrease in the naïve T cell numbers	NA
Maarschalk-Ellerbroek et al., 2012, Netherlands (25)	9/61	25:36	27 (14-43)	At diagnosis, 3 patients had cytopenia (AIHA/ITP), and it increased to 9 at follow-up (median 7 years); splenomegaly seen in 8 patients; low switched memory B cells associated with autoimmunity, splenomegaly and granulomas	NA
Arshi et al., 2016, Iran (26)	21/47	1:1	27 (4-63)	ITP was the commonest manifestation (26%) followed by AIHA (15%) and pernicious anaemia (4%)Autoimmunity occurred in older age group (mean 14.2 years) and was associated with parental consanguinity (57%)	IVIg in all and splenectomy in 3 patients with ITP
Patuzzo et al., 2016, Italy (27)	10/10	1:4	44.8 (±12)	Patients with CVID and AITP had a higher percentage of CD21low cells	NA
Arduini et al., 2016, Ireland (28)	2/23	13:10	22-82	1 patient had AIHA, ITP and neutropenia; 1 patient had pernicious anaemiaPeripheral mucosal-associated invariant T cell activation is a feature of CVID and depletion of these cells is particularly associated with complications including autoimmunity	NA
Çalişkaner et al., 2016, Turkey (29)	3/25	12:13	36.6 (± 13.4)	3 patients had ITP (2 had splenomegaly and 1 required splenectomy)	IVIg and steroid
Almejun et al., 2017, Argentina (30)	5/25	12:13	11.3 (4-16.1)	Severe altered somatic hypermutation in addition to low switched memory B cells has a correlation with autoimmunity, splenomegaly and granulomas	NA
Feuille et al., 2017, USA (USIDNET Registry) (8)	101/990	52:49	16 (10-31)	The most common autoimmune cytopenia was ITP (N = 73), followed by haemolytic anaemia (N = 45), and autoimmune neutropenia (N = 10); There was no significant difference in the age at diagnosis, gender, and baseline Ig values between the group with autoimmune cytopenia and those without cytopenia; autoimmune cytopenia group was more likely to have lymphoproliferation, granulomatous disease, lymphomas, hepatic disease, interstitial lung diseases, enteropathy, and organ-specific autoimmunity	NA
Guffroy et al., 2017, France (31)	16/473	1.7:1	17 (4-63)	Frequency of neutropenia 3.4%.16 patients had neutropenia and 11 of them were AINFive patients died during the follow-up (11 years) with an increased percentage of deaths in patients with neutropenia	Specific treatment for neutropenia was in general not administered, except in 3 patients who received G-CSF
Alkan et al., 2018, Turkey (32)	2/12	7:5	11.6 ( ± 3.7)	2 patients had Evans syndrome and splenomegalyBoth patients with cytopenias were diagnosed after 10 years	NA
Ghorbani et al., 2019, Iran (33)	18/220	1.2:1	9.5 (3.9-18.25)5 (1.8-10)**	Frequency of neutropenia was 8.1%; Candida infection and septicaemia were significantly higher in neutropenic patients; the most prominent clinical phenotypes of CVID patients with neutropenia were polyclonal lymphocytic infiltration and autoimmunityThe mortality rate in neutropenic patients was higher than in patients without neutropenia (61.1 vs. 25.2%, p=0.004)	IVIg and prophylactic antibiotics for neutropeniaG-CSF and splenectomy were considered in 1 and 2 patients respectively
Mormille et al., 2021, Italy (10)	17/95	9:8	24-76	The most common autoimmune manifestation was cytopenia (17.8%); the most common cytopenia was immune thrombocytopenia, reported in 10 out of 95 patients (10.5%), followed by autoimmune haemolytic anaemia (n=3, 3.1%) and autoimmune neutropenia (n=3, 3.1%); almost all patients with autoimmune cytopenia had splenomegaly (15 out of 17; 88%)There was no statistically siginificant difference in CD3+, CD8+, CD4+CD25highCD127low T reg, CD19, CD19hiCD21loCD38lo, and follicular T helper cells in CVID patients with or without autoimmune manifestations	IVIg and steroid1 patient underwent splenectomy

NA, not available; CVID, common variable immunodeficiency; IVIg, Intravenous immunoglobulin; G-CSF, Granulocyte colony stimulating factor.

^#^x: no. of autoimmune cytopenia patients, y: total no. of CVID patients.

*97 patients had autoimmune manifestations (exact number of patients with autoimmune cytopenic not reported).

**Neutropenic patients with CVID.

### Immune Thrombocytopenic Purpura

Of the various autoimmune cytopenia in CVID, ITP is the most frequently reported manifestation by several authors (8–12, 14–17, 21, 24–26) Initial studies from United Kingdom (UK) and United States of America (USA) reported that autoimmune cytopenia is more common in females with CVID (11, 12). It was also opined that females tend to have a later onset of disease as compared to males. These studies, however, did not classify the effect of autoimmunity on different cell lineages and included cytopenia as a whole. In the USA cohort, it was observed that both serum IgA and IgM were higher in females and this finding was postulated to be a risk factor for autoimmunity (12). Another study by Kokron et al. found that although women had late-onset autoimmunity, the overall morbidity and mortality remained similar for both genders (14). This observation is similar to most other studies over last two decades that have shown that there is no gender predisposition to autoimmunity in patients with CVID (**Table 1**).

Proportion of patients with CVID who develop ITP has been reported to vary from 7.4 to 19% (**Table 1**). These differences could be attributed to the study design or hitherto unknown genetic differences in different ethnicities. Studies prior to 2000 have shown that most patients with CVID with ITP had mild symptoms (11, 12). Even in patients with clinically significant thrombocytopenia, splenectomy was not considered as a therapeutic option by most treating physicians. However, recently it has been noted that splenectomy may be considered in refractory cases of ITP. Splenectomy was not found to increase overall morbidity and mortality, provided that these patients were continued on regular intravenous immunoglobulin (IVIg) replacement (10, 12, 19, 25, 26).

At times, ITP may be the first and only symptom of CVID. A French study in 2004 included 21 patients with ITP who were also diagnosed to have CVID (15). Of these, most patients (62%) had delay in diagnosis of CVID (more than 6 months after the diagnosis of ITP). Only 19% patients were diagnosed to have CVID before the diagnosis of ITP. Another study from USA in 2005 has reported that most patients (54%) had ITP as the first manifestation of CVID (16). A large multicentric study from Europe also reported that presence of ITP often delays the diagnosis of CVID (21). Thus, it may be suggested to check serum immunoglobulins in all patients with ITP who often report to the haematology clinic.

### Autoimmune Haemolytic Anaemia

Following ITP, the other most common autoimmune cytopenia in CVID has been reported to be AIHA (5). As in cases of ITP, women tend to have a later onset of disease, although the overall morbidity and mortality remained the same between the 2 genders (21). There is a wide variation in proportion of patients with CVID who have been reported to develop AIHA (between 1 to 15%) (**Table 1**). In one study involving 326 patients with CVID, cytopenia was seen in 11% (*n* = 35): 9 had AIHA, and 11 had Evans syndrome (16). Most patients developed autoimmune cytopenia before or concurrent with the diagnosis of CVID. A similar observation has also been reported by several other authors. It may also be suggested to test for serum immunoglobulins in all patients who have AIHA.

Polyautoimmunity has been reported in as high as one-third of all patients with CVID who have autoimmune cytopenia (34). Although various other organ systems may be involved, the commonest association of AIHA is with ITP (Evans syndrome) (34). A multicentric study by Wehr et al. observed that Evans syndrome was seen in 11% patients with CVID who had autoimmune cytopenia (21). Besides, both AIHA and ITP may occur concomitantly with autoimmunity in other organ systems including gastrointestinal, endocrine, rheumatological and dermatological. A recent meta-analysis has shown that haematological autoimmunity coexists with gastrointestinal and rheumatological autoimmunity in 3.1% and 2.1% patients respectively (34).

### Autoimmune Neutropenia

There may be several causes of neutropenia in CVID. These include infection/sepsis induced, drug related, sequestration by spleen, autoimmunity or paradoxical neutropenia following IVIg infusion (31, 33). Most published literature on neutropenia in CVID is in the form of case reports and case series. These studies have reported neutropenia in <1% to 4% of all CVID patients (**Table 1**). An Iranian study observed neutropenia in 8.1% of all patients with CVID (33). However, in this cohort, all causes of neutropenia were included.

Similar to ITP and AIHA, there is no significant gender difference in the proportion of patients who develop AIN. However, in contrast to other forms of autoimmune cytopenia, patients with AIN are diagnosed early and the diagnosis of AIN rarely antedates the diagnosis of CVID (31, 33).

Polyautoimmunity is also commonly seen with AN and the most frequent associations are with ITP and AIHA (19). Ghorbani et al. also reported rheumatoid arthritis, vitiligo and autoimmune hepatitis in association with AIN (33).

There has been a frequent association of infections with AIN. However, whether this infection causes neutropenia or neutropenia per se is because of autoimmunity and is contributing to infections, remains contentious. In a study from Iran, fungal infections such as candidiasis and pancytopenia (27.5%) were observed more commonly in patients with neutropenia (33). Another study from French DEFI cohort reported that patients with AIN have unusual opportunistic infections such as *Pneumocystis sp*, deep mycotic infections, cryptosporidium, aspergillosis and cytomegalovirus colitis (31).

Patients with CVID and AIN have been reported to have poor prognosis. Ghorbani et al. reported a higher frequency of deaths (61.5%) in their cohort of patients with CVID who had AIN (33). Another study reported an eight-year overall survival rate of 50% in patients with AIN as compared to 87.5% survival rate in non-neutropenic patients (31). Thus, neutropenia in patients with CVID warrant prompt investigation and initiation of appropriate therapy as it may have an impact on overall mortality.

### Pernicious Anaemia

Pernicious anaemia has been reported to be the least common amongst the various autoimmune cytopenia associated with CVID (**Table 1**). There are no large studies on PN in CVID. In a recent meta-analysis, the prevalence of PN was reported to be 2.4% (95% CI) (34).

Although PN has been described in literature since the 1840s, however, PN in the context of CVID was first described in 1969 (35). Most authors have defined classical PN as the presence of: “(1) Haemoglobin concentration < 13 g/dL for men and <12 g/dL for women, (2) red blood cell’s mean corpuscular volume ≥ 120 fL, (3) low levels of serum vitamin B_12_, (4) gastric body mucosal atrophy, and (5) auto-antibodies to intrinsic factor and/or to gastric parietal cells” (36). However, PN in CVID has been described in association with achlorhydria, atrophic gastritis, absence of intrinsic factor, absence of antibodies to gastric parietal cells and intrinsic factor, and malabsorption of vitamin B_12_ (37, 38). This entity may, at times, be difficult to differentiate from classical pernicious anaemia. However, PN in CVID usually occurs early and has low to absent autoantibodies, and presence of atrophic gastritis without plasma cell infiltrate in the lamina propria (38). The pathogenesis remains unexplained although it has been hypothesized that this subset of patients with CVID may have additional T-cell defects (37).

## Pathogenesis of Autoimmune Cytopenia in CVID

Mechanism of autoimmunity in CVID remains an enigma. Both innate and adaptive arms of the immune system have been found to play a role in the pathogenesis of autoimmunity in CVID including autoimmune cytopenia (3). **Table 2** lists the salient findings of various studies that have reported immune abnormalities associated with autoimmune cytopenia in patients with CVID.

**Table 2:** Review of studies that have reported the immunopathogenesis of autoimmune cytopenia in CVID.

Author, year, country	Title	N	Technique used	Salient Features
G. Azizi et al., 2017; Iran (3)	Autoimmunity and its association with regulatory T cells and B cell subsets in patients with common variable immunodeficiency	72	Flowcytometric evaluation of T and B cell compartment	Higher transitional MZ B cells in patients with CVID with autoimmune cytopeniaLower percentage of naive and non-class-switched memory B cells were seen in patients with CVID with autoimmunityPatients with CVID with multiple autoimmune syndromes had higher level of CD3+ T cells, CD4+ T cells and CD21low B cells and lower number of T regs and naïve B cell when compared with patients with CVID with one autoimmune syndrome
Warnatz et al., 2002; Germany (39)	Severe deficiency of switched memory B cells (CD271IgM2IgD2) in subgroupsof patients with common variable immunodeficiency: a new approach toclassify a heterogeneous disease	38 (30 CVID; 22 HC)	Flowcytometry	Reduced class switched memory B cells (<0.4%) and increased CD21low B cells (>20%) in patients with autoimmune cytopenia
E. Kofod-Olsen et al; 2016., Denmark (40)	Altered fraction of regulatory B and T cells is correlated with autoimmune phenomena and splenomegaly in patients with CVID	34; 11(HC)	Flowcytometry:Intracellular IL-10 expression analysisIntracellular FoxP3 expression analysisT cell suppression assay	Pronounced Reduction in Tregs in patients with CVID with autoimmunityrTregs (resting) were significantly reduced in the autoimmunity grouppatients had a significant reduction in CTLA-4 expression in all subsets except the rTregsSignificantly high expression of pro-B10 cells in autoimmunity group
Genre et al., 2009; Brazil (41)	Reduced frequency of CD4+CD25HIGHFOXP3+ cells and diminished FOXP3 expression in patients with Common Variable Immunodeficiency:A link to autoimmunity?	33, 30(HC)	Flow cytometric analysisRT PCR of FOXP3	Decrease of absolute CD4+ lymphocytes numberslower frequency of CD4+CD25HIGHFOXP3+ cells in patients with AI with CVID than without AIReduced FOXP3 mRNA levels in Tregs of patients with CVID (Higher Reduction in AI+CVID group)
Tahiat A et al., 2014; Algeria (42)	Common variable immunodeficiency (CVID): clinical and immunological features of 29 Algerian patients	29	Flowcytometry	Decreased circulating B (54.2%) and T CD4+ (41.7%) cells and inversion of the CD4/CD8 ratio (70.8%). Patients with decreased circulating B and T CD4+ cells were significantly more likely to have auto-immune cytopenias and lymphoproliferative disease.
Mouillot G, et al., 2010 (23);	B-Cell and T-Cell Phenotypes in CVID Patients Correlate with the Clinical Phenotype of the Disease.	313; 50(HC)	Flowcytometry	Reduced smB cells, Increased CD21^low^ B cellsSignificant reduction in activated T cells (CD4^+^and CD95^+^ T cells) (AC>IO group)Reduced CD4+ and HLADR^+^ T cells and Tregs
Romberg et al., 2019 (43)	CVID patients with autoimmune cytopenias exhibit hyperplastic yet inefficient germinal centre responses	14 CVID+AIC and 4 CVID-AIC patients.	Flowcytometry *In Vitro* T suppression activity, Lymph node staining and RT PCR	CVID+AIC patients displayed irregularly-shaped, hyperplastic germinal centres (GCs), whereas GCs were scarce and small in CVID-AIC patients evidenced by an increase in circulating T follicular helper cells, which correlated with decreased regulatory T cell frequencies and function

MZ, Marginal Zone; RT PCR, Polymerase chain reaction; Tregs, regulatory T cells; HC, Healthy controls; rTregs, Resting regulatory T cell; aTregs, Activated regulatory T cells; CTLA4, Cytotoxic T lymphocyte associated protein 4; pro-B10, regulatory B cells; AI, Autoimmunity; smB, Switched memory B cells; AIC, Autoimmune cytopenia; GC, Germinal Centre; IO, Infection Only.

## Role of Dysregulated B Cells in CVID Associated Autoimmune Cytopenia

Autoimmunity in patients with CVID is a complex pathophysiological mechanism as it represents a state of overreactive immune system in an otherwise immunocompromised host. Impairment in the development and function of B cells is a hallmark of CVID. Most patients with CVID have normal peripheral B cell counts and reduced CD27^+^ memory B cells with severely impaired capacity to produce antibodies. A proportion of patients with CVID, however, tend to produce autoantibodies against self-antigens (44).

**Figure 1:**
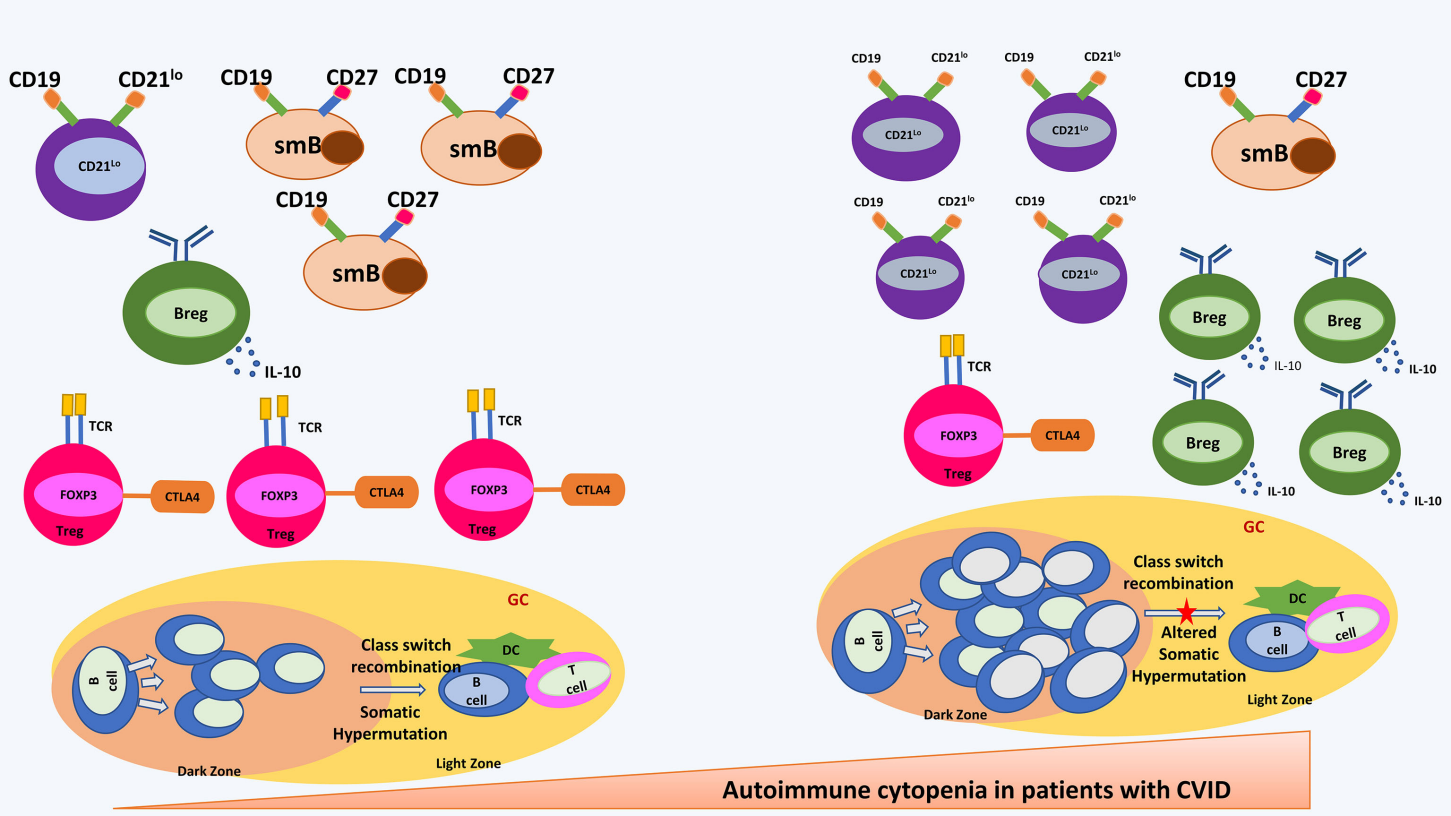
shows most important immune abnormalities that have been reported in patients with CVID with autoimmune cytopenia. Shown in the right panel are an increased CD21^lo^ B cell and B regulatory cells; decreased switched memory B cells and T regulatory cells; hyperplastic germinal centre and altered somatic hypermutation. CD21^lo^: CD21^-/LOW^ B cells; smB, switched memory B cells; Breg, B regulatory cells; Treg, T regulatory cells; DC, Dendritic Cells; GC, germinal centre.

Studies have shown that development of autoimmune cytopenia in CVID is linked to a lower efficacy of the self-tolerance mechanisms thereby leading to an altered immune-regulation (8). It has been observed that patients with CVID with autoimmune cytopenia may have characteristic abnormalities in the B cell immunophenotyping. These patients have been reported to have significantly reduced numbers of CD19+ B cells as compared to patients with CVID who do not develop autoimmune cytopenia (45). The most striking abnormality, however, is the expansion of an unusual population of B cells that lack complement receptor 2 (CR2/CD21) [CD21^lo^ B cells] (45). CD21^lo^ B cells represent a pool of autoreactive B cells. Autoreactive B cells are generated during random process of V(D)J recombination. Autoreactive B cells are generally silenced by 3 main mechanisms: deletion, receptor editing, and anergy. Receptor editing and deletion results in central tolerance that omits autoreactive immature B cells. However, a small percentage of autoreactive B cells escape the bone marrow and remain in periphery where anergy renders them unresponsive to antigenic stimuli (46). Important immune abnormalities have been illustrated in **Figure 1**.

CD21^lo^ B cells manage to escape the central B-cell tolerance and remain in the periphery in an unresponsive stage. Low proportion of these cells are also present in healthy individuals. CD21^lo^ B cells have short life span and are usually eliminated in normal individuals. However, various studies have shown an expansion of CD21^lo^ clones of autoreactive B cells in patients with systemic lupus erythematosus (SLE), CVID and rheumatoid arthritis (RA) (47). Factors that favour maintenance and survival of the autoreactive and unresponsive CD21^lo^ B cells in the peripheral circulation of patients with RA and CVID are unknown. It has been suggested that elevated concentrations of B cell activating factor (BAFF) in the serum of patients with CVID lead to inhibition of removal of anergic CD21^lo^ B cells from periphery (48). Presence of anergic CD21^lo^ B cells with low avidity to bind to self-antigens pose a major risk of development of autoimmunity. Murine studies have shown that inactive CD21^lo^ B may overcome the state of anergy during infection where cross-reactive antigenic epitopes present on infectious agents stimulate anergic B cells *via* innate immune ligands (49).

Isnardi et al. studied the pool of CD21^lo^ B cells in patients with CVID and RA. CD21^lo^ B cells were found to be elevated in these patients. It was further observed that these CD21^-/lo^ B cells are closer to the naïve B cell population (in comparison to the isotype switched CD21^lo^ B cells seen in healthy individuals) and express germline B-cell receptor (BCR) repertoire that is rich in autoreactive clones. Immunofluorescence assay showed that CD21^lo^ B cells expressed antinuclear antibodies (speckled nuclear and nucleolar pattern) and also expressed autoantibodies against several cytoplasmic structures. CD21^lo^ B cells do not get activated and do no proliferate through BCR and CD40 co-stimulation and showed impaired calcium-mediated signalling. This results in inactivation of a few activation markers on B cells upon BCR triggering. Impaired activation has been linked to impaired proliferation of B cells to antigenic stimuli suggesting their unresponsive stage. The transcriptome analysis of CD21^lo^ B cells revealed up-regulation of several genes implicated in the inhibition of B-cell activation, proliferation, and survival and the downregulation of B-cell activating genes, suggesting an inhibitory gene signature. These results were further confirmed using flow cytometry assays that suggested downregulation of receptors that favour B cell survival and upregulation of receptors that favour B cell inhibition. In addition, the survival potential of CD21^lo^ B cells was compared with that of CD21^+^ B cells and it was found that CD21^lo^ B cells were prone to die by apoptosis suggesting they have a shorter half-life (47).

Warnatz et al. classified CVID patients with low CD27**^+^** B cells into 2 groups based on proportion of CD21**^lo^** B cells. Group Ia had more than 20% CD21**^lo^** B cells and these patients were found to be more susceptible to develop autoimmune cytopenia (and not the other autoimmune manifestations) (39).

The French DEFI group screened 311 patients with CVID and divided them (based on the clinical manifestations) into non infection (NI), autoimmunity (AI) and autoimmune cytopenia (cy) group. Absolute numbers of B and T cells were low but comparable among the 3 groups. However, a significant association between CD21^lo^ B cells and autoimmune cytopenia was reported. Percentage increase in CD21^lo^ B cells was comparable in NI and AI group while it was significantly high in the cy group. This suggests that higher proportion of CD21^lo^ B cells in patients with CVID correlate specifically with an increased risk of autoimmune cytopenia (23).

Role of pro B10 cells (also known as regulatory B cells [Bregs], identified by the production of IL-10 cytokine) has been reported in a number of immune mediated disorders, such as RA and ITP. Olsen et al. reported that pro B10 cells were increased in patients with CVID who developed autoimmunity and splenomegaly whereas patients with CVID without autoimmunity displayed only a modest increase in these cells (40). The underlying mechanistic link between elevated pro-B10 cell levels and autoimmunity in CVID patients is not clear at present. However, contrasting results have been reported in murine models and patients with RA. Proportions of Bregs have been reported to be reduced and inversely related to disease severity in patients with RA. This fraction of B cells has been reported to be increased in patients with ITP and patients with chronic hepatitis. It has been hypothesized that increase in Bregs in patients with CVID suggest a compensatory mechanism wherein the Bregs expand to compensate for reduced level of regulatory T cells (Tregs) and this effect seems to be more pronounced in patients with autoimmunity and splenomegaly (43).

Romberg et al. compared the germinal centre (GC) responses of patients with CVID with autoimmune cytopenia (CVID+AIC) and CVID patients with autoimmunity other than cytopenia (CVID-AIC). Irregularly shaped and hyperplastic germinal centres along with increased number of circulating T follicular helper cells were observed in CVID+AIC group while GC structure in CVID-AIC group were found to be small and circular. CVID+AIC cohort had higher CD19hiCD21-/lo B cells compared to CVID-AIC cohort, CD27+IgG+ memory B-cell population and IgA+ B cells were reduced in CVID+AIC group (43).

The study also evaluated somatic hypermutation (SHM) in CD27+IgG+ memory B-cells. Patients with CVID were found to have lower SHM frequencies in heavy chain variable regions (VH) than controls. CVID+AIC patients showed least SHM (7.5 mutations per VH segment) as compared to 15.1 in CVID-AIC group and 18.6 in heathy controls. VH4-34 gene segment was identified in 9.9% of CVID+AIC IgG transcripts while this segment was rarely seen in CVID-AIC group and healthy controls. VH4-34-encoded antibodies have been found to be autoreactive as they bind the conserved I/i carbohydrate self-antigens expressed in red blood cells and other hematopoietic cell lineages (43).

Yu et al. have also reported that patients with autoimmunity with CVID had significantly reduced switched memory B cells (50).

## Role of Dysregulated T Cells in CVID Associated Autoimmune Cytopenia

Role of T cell compartment in the development of autoimmune cytopenia in patients with CVID has also been reported by several authors. Disturbed T cell homeostasis underlies the pathogenesis in one third of CVID patients with autoimmune manifestations. These include alterations in number of CD4, CD8 T cells, memory T cells, regulatory T cells, and altered expression of transcripts essential for regulatory T cells functioning.

The French DEFI group study reported reduced number of switched memory B cells and naïve CD4^+^ T cells in CVID associated autoimmune cytopenia. This reduction was accompanied by activated phenotype with an increased expression of HLA-DR and CD95 markers on CD4^+^ T cells. Patients with other autoimmune manifestations did not show this T and B cell phenotype (23).

In another study on 29 patients with CVID, abnormality in T and B cell phenotypes was detected in 75% cases, mostly reduced circulating B cells (54.2%) and CD4+ T (41.7%) cells. There was inversion of CD4/CD8 ratio (70.8%). Patients with decreased circulating B and CD4+ T cells were significantly more likely to have auto-immune cytopenias and lymphoproliferative disease (42).

It has also been reported that patients with CVID with autoimmune manifestations have significantly reduced proportion of CD8+ T cells (51).

Herrera et al. compared the absolute numbers of T, B and NK cells among patients with CVID. Lymphocyte profiles were compared between patients with CVID with autoimmunity and patients with CVID without autoimmunity and healthy controls. CD4+ T cell numbers in patients with CVID without AI were significantly lower compared with the control group. Patients with CVID with AI had increased CD4+CD45RO+ memory T cell populations compared with healthy controls (45).

Bateman et al. reported that naïve CD4^+^ and CD8^+^ T cell numbers were significantly reduced in patients with CVID especially in association with autoimmune cytopenia. Further, within CD4+ T cell compartment, there was reduction in CD4^+^CD45RA^-^CCR7^+^ central memory T cells in autoimmune cytopenia group. In CD8+ T cells, CD8^+^CD45RA^-^CCR7^-^ effector memory T were reduced in patients with CVID with organ specific autoimmunity and increased in patients with CVID with autoimmune cytopenia.

Enumeration of early differentiation stages of CD4+ and CD8+ T cells defined by co-expression of CD27/28 molecules revealed reduced numbers in autoimmune cytopenia and organ specific autoimmunity subgroups of patients with CVID. Reduction in the population of CD4+ and CD8+ T cell numbers was not accompanied by an increase in the numbers of recent thymic emigrants, suggesting a lack of replenishment of the lymphocyte pool from thymus (51).

In addition to these abnormalities various studies have highlighted the role of Tregs in patients with CVID with autoimmune manifestations. Tregs play pivotal role in limiting the persistent immune activation. Reduced counts along with impaired suppressive capacity of Tregs has been reported in literature

Freiburg classification differentiates patients with CVID into groups Ia and Ib with significantly lower percentages of Tregs compared to patients in group II and healthy controls. Autoimmune disease was found to be significantly higher in group Ia (52).

Horn et al. reported reduced number of Tregs in patients with CVID with immune cytopenia and granulomatous diseases (53).

A negative correlation between reduced Treg numbers and presence of autoimmunity in patients with CVID with AI has also been reported (45) (51).

Olsen et al. reported altered proportions of regulatory lymphocytes in patients with CVID. Patients with autoimmunity had reduced levels of resting Tregs and activated Tregs predominantly seen in patients with autoimmunity and splenomegaly. The impaired functioning of activated Tregs was further indicated by reduced expression of cytotoxic T lymphocyte antigen 4 (CTLA-4) on its surface (40).

Reduced number of Tregs and an increase in T follicular helper CD4+ cells has been reported in patients with CVID with autoimmune cytopenia (41). Genre et al. reported compromised homeostasis of Tregs in a subset of patients with CVID with autoimmunity. Flowcytometry revealed reduced proportion of CD4^+^CD25^HIGH^FOXP3^+^ Tregs in patients with CVID with autoimmunity as compared to patients with CVID without autoimmunity. Forkhead box P3 protein (FOXP3) mRNA (messenger ribonucleic acid) expression was also found to be reduced in patients with CVID compared to healthy controls and the reduction was more pronounced in patients who had autoimmune cytopenia (41).

Yu et al. reported Tregs dysfunction in patients with CVID with autoimmunity. Switched memory B cells and Tregs were found to be low along with reduced ability to suppress proliferation of autologous and allogenic CD4+ effector cells in patients with CVID with autoimmunity when compared with patients with CVID without autoimmune disease, healthy controls and disease control (patients with X-linked agammaglobulinemia). The key proteins involved in functioning of Treg, including FoxP3, Granzyme A, XCL1 (lymphotactin), pSTAT5 (phosphorylated signal transducer and activation of transcription-5 protein), and GITR (glucocorticoid induced tumor necrosis factor receptor related protein) were found to be significantly reduced in patients with CVID with autoimmunity. Results suggest that these proteins may be involved in Treg-mediated autoimmunity in patients with CVID (50).

## Dysregulation of Innate Immune System in CVID Associated Autoimmune Cytopenia

Although defects in innate immunity have been reported in patients CVID, their correlation with autoimmunity has not been investigated in detail. Taraldsrud et al. and Sharifi et al. studied the role of Toll-like receptors (TLR) in the pathogenesis of CVID (54, 55). It was suggested that defective TLR7, TLR8, and TLR9 signalling may lead to dysregulation of self-tolerance and expansion of auto-reactive B cells.

Rezaei et al. measured various cytokines, especially type I interferons (IFN), in patients with CVID. It has been postulated that increased IFN-α/β may result in dysregulation of peripheral tolerance by activating immature dendritic cells (56). They may also lead to activation of autoreactive T cells, that in turn would increase autoreactive B cells and subsequent autoimmunity. However, evidence is still lacking and further studies are needed on this aspect (57).

## Pathophysiology of Non-CVID Associated Autoimmune Cytopenia

Autoimmune cytopenia may also be seen in several other disorders such as inborn errors of immunity (e.g. Wiskott-Aldrich syndrome, autoimmune lymphoproliferative syndrome, X-linked lymphoproliferative syndrome, severe combined immunodeficiency and complement defects), acquired causes such as (lymphoproliferative disorders, malignancies, systemic lupus erythematosus [SLE], drugs, infections and complication of organ or hematopoietic stem cell transplant). The pathophysiology of autoimmune cytopenia in several of these disorders especially those associated with inborn errors of immunity is more complex and similar to the mechanisms associated with CVID. On the other hand, the pathophysiology of autoimmune cytopenia in acquired disorders such as SLE or drug induced cytopenia is primarily associated with generation of auto-antibodies [e.g. autoantibodies against receptors on the platelet surface, GPIb/IX complex (vonWillebrand factor receptor) and the GPIIb/IIIa receptor (collagen/fibrinogen receptor) may be associated with autoimmune thrombocytopenia in SLE and antibodies against red blood cells may be associated with warm-reactive (W-AIHA), cold-agglutinin (C-AIHA), or paroxysmal cold haemoglobinuria (PCH)] (58, 59).

Autoimmune cytopenia in patients with autoimmune lymphoproliferative syndrome is primarily due to defective apoptosis of lymphocytes mediated through the Fas/Fas ligand pathway (60). On the other hand, pathophysiology of autoimmune cytopenia in context of complement defects is associated with defective clearance of apoptotic bodies and formation of immune complexes (59).

### Genetic Link to Autoimmune Cytopenia in CVID

Monogenic defects have been identified in a small proportion of patients with CVID. These monogenic defects may have an important pathophysiological link with autoimmune cytopenia.

*TNFESF13B* gene encodes for TACI (transmembrane activator and calcium-modulating cyclophilin ligand interactor), a member of tumour necrosis factor receptor superfamily expressed on B cells. TACI has been found to play important role in the B cell development. Monoallelic heterozygous and biallelic (compound heterozygous and homozygous) defects in the gene encoding for TACI have been reported to cause CVID. However, there are speculations that TACI defects are diseases modifying rather than disease causing because several healthy individuals have been reported to have same defect but do not develop any clinical manifestations. A recent study from Greece reported that monoallelic defects in TACI may act as susceptibility or disease modifying factor in the pathogenesis of CVID. It was, however, observed that patients with CVID with TACI defects had significantly higher risk of autoimmune cytopenia as compared to patients with CVID without any TACI defect (61). In addition, studies have shown that TACI plays an important role in central B cell tolerance and defects in TACI lead to impaired central B cell tolerance leading to an increased production of autoreactive B cells (62). It is intriguing to note that patients with CVID with TACI defects and not the carriers of TACI defects are more prone to develop autoimmunity. The likely explanation for this is that patients with CVID have defect in peripheral B cells tolerance while this is not seen in healthy individuals with TACI defects. As a result, patients with CVID with TACI defects are unable to compensate for loss of central B cell tolerance which is well compensated in healthy carriers of TACI defect. Moreover, the heterozygous monoallelic variants rather than biallelic variants are more likely to produce autoimmunity. This is because a more profound defect in TLR pathway defects in patients with CVID with biallelic variants in TACI provides protection against development of autoimmunity even though autoreactive B cells are also increased in patients with biallelic variants in TACI (63).

Tumor necrosis factor receptor superfamily member 13C (TNFRSF13C) encodes for BAFF-R (B-cell activating factor receptor) that functions as a pro-survival factor for B cells. Variants in BAFF-R lead to arrest of developing B cells at immature/transitional B cells stage. Similar to the TACI defect, the variants in BAFF-R may possibly be disease modifying rather than disease causing. A few patients with BAFF-R deficiency have been reported to develop autoimmune manifestations (6). The exact pathogenesis is not known but could be related to elevated serum BAFF levels because of BAFF-R deficiency (64). BAFF, which belongs to the TNF-ligand family, plays crucial role in B cell development, maintenance of auto-reactivity, and homeostasis (65, 66). Plasma BAFF levels have been found to be elevated in autoimmune disorders including SLE (67), RA (68), Sjögren syndrome (SS) (69). BAFF has also been found to be elevated in active ITP and levels normalise during remission (70). Elevated levels of BAFF promote the survival of auto-reactive B cells (71) and may lead to autoimmune cytopenia in CVID.

Inducible T cell costimulator (ICOS), a member of CD28/CTLA-4 family, plays important role in regulating T cell responses. ICOS deficiency was the first identified genetic defect in patients with CVID. ICOS ligand is expressed in monocytes, dendritic cells and B cells. In addition to hypogammaglobulinemia and recurrent infections, these patients have also been reported to develop autoimmune manifestations especially autoimmune neutropenia (72, 73) (74). The exact pathophysiology of autoimmunity in ICOS deficiency is not known. However, it has been suggested that decreased production of IL-10 and decreased expression of CTLA-4 in patients with ICOS deficiency is responsible for autoimmune manifestations (72). In the original description of ICOS deficiency in context of CVID, patients with autoimmune neutropenia were detected to have IgG antineutrophil antibodies, suggesting an ICOS independent class switch in these patients (73).

Lipopolysaccharide-responsive and beige-like anchor (LRBA) protein encoded by the *LRBA* gene, is a critical protein involved in the expression and intracellular trafficking of CTLA4 protein. Costimulatory signal between T cells and antigen presenting cells (APCs) using CD28 (on T cells) and CD80/86 on APCs is crucial in the activation of T cells. CTLA-4 has higher affinity for CD80/86 and outcompetes CD28 in binding to CD80/86, CTLA-4, therefore, constitute an important immune check-point by preventing overactivation of T cells. CTLA-4 is an important mechanism by which Tregs exert their inhibitory effect on activated T cells. Patients with homozygous or compound heterozygous variants in *LRBA* gene and heterozygous variants in *CTLA-4* gene fail to express CTLA-4 protein on surface and have been reported to develop CVID phenotype with autoimmunity, lymphoproliferation and inflammation (75). LRBA deficiency is one of the commonest genetic defects identified in patients with CVID (76).

Patients with LRBA deficiency and CTLA-4 haploinsufficiency present with a broad and overlapping clinical phenotype. Most common autoimmune manifestation in both these disorders include autoimmune cytopenia (seen in more than 2/3^rd^ of all cases) (71, 73). LRBA deficiency and CTLA-4 haploinsufficiency leads to a normal or elevated number of Treg cells in the circulation. However, the Treg cell functions are impaired.

It has also been reported that monogenic defects may be identified in more that 2/3^rd^ of all patients with Evans syndrome (autoimmune haemolytic anaemia and thrombocytopenia) especially defects in *LRBA* and *CTLA-4* gene. Other genetic defects reported in patients with Evans syndrome include heterozygous loss of function mutation in *TNFRSF6* gene, *CBL* gene and *ADAR1* gene; heterozygous gain of function mutations in *STAT3* gene and *PIK3CD* gene; and compound heterozygous mutations in *RAG1* gene. In addition, somatic mutations in *TNFRSF6* and *KRAS* genes and possibly pathogenic variants in several other genes were also reported. Patients with Evans syndrome who had a monogenic defect were more likely to have hypogammaglobulinemia and lymphoproliferation as compared to the patients with Evans syndrome who had no monogenic defects (77, 78).

BCR complex is composed of CD19, CD21, CD81 and CD225. Monogenic defects in CD19, CD21 and CD81 have been reported to lead to CVID phenotype. Of these, patients with CD19 and CD81 deficiency have also been reported to develop autoimmunity and autoimmune cytopenia have been reported in patients with CD81 deficiency (79). BCR complex along with toll like receptor mediated signalling is essential for removal of autoreactive B cells. As a result, patients with defect in components of BCR may be predisposed to develop autoimmune cytopenia.

Patients with activated Phosphoinositide 3-kinase (PI3) δ syndrome (APDS) present with a CVID or hyper IgM phenotype with predominant clinical manifestation of autoimmunity (especially autoimmune cytopenia) and lymphoproliferation (80, 81). APDS is caused by a gain of function mutation in the *PIK3CD* gene that encodes for catalytic subunit (p110δ) of PI3Kδ [APDS 1] or loss of function mutations in *PIK3R1* gene that encodes for regulatory subunit (p85α) of PI3Kδ [APDS2] (82). The end result of these molecular defects is an overactivation of the mammalian target of rapamycin (mTOR) pathway that leads to cell survival, cell proliferation and inhibition of apoptosis. Development of autoimmunity in APDS is a complex mechanism. B cell apoptosis in the germinal centre is an important mechanism to eliminate auto-reactive B cells. This mechanism along with B cell hyperactivation and enhanced proliferation may lead to autoimmunity including autoimmune cytopenia (83).

Heterozygous pathogenic variants in the *NFKB2* lead to a CVID phenotype along with a distinct pattern of autoimmune manifestations. Unlike most patients with CVID wherein autoimmune cytopenia is the most common autoimmune manifestations, this is not the most common autoimmune manifestation in patients with NFKB2 gene mutation (84). Autoimmunity in patients with haploinsufficiency of *NFKB2* is more likely to be T cell driven and unlike the mechanism of autoimmunity in other forms of genetic defects causing CVID, there is no significant role of autoantibodies. *NFKB2* also has important role in central tolerance. *NFKB2* signalling is important for the development of medullary thymic epithelial cells and regulation of autoimmune regulator (AIRE). As a result of haploinsufficiency of *NFKB2*, there is loss of central tolerance mechanism leading to accumulation of auto-reactive T cells.

**Figure 2:**
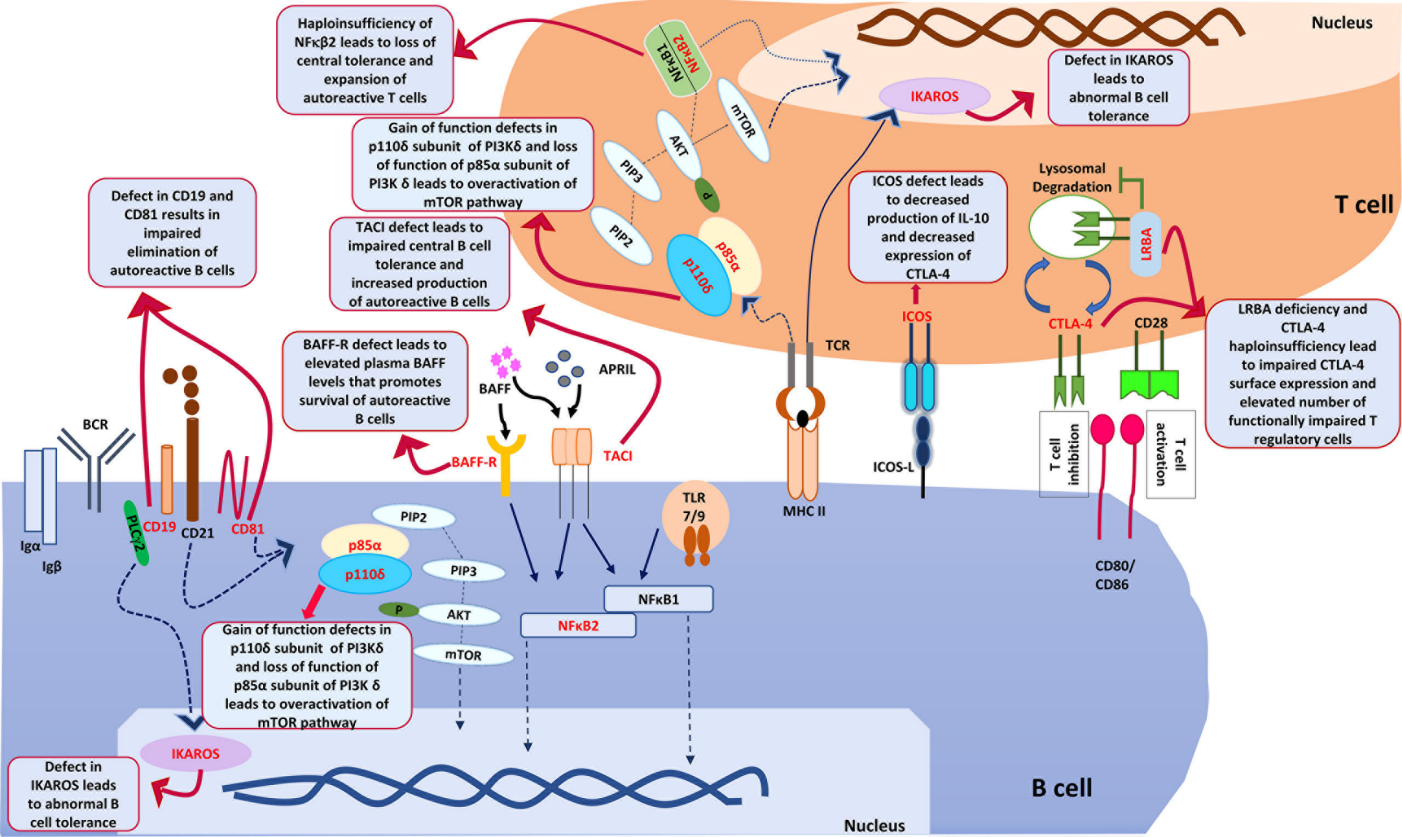
shows various genes and downstream pathways that are involved in pathogenesis of autoimmune cytopenia in patients with CVID. ICOS, Inducible T cell costimulator; ICOS-L, Inducible costimulator ligand; CTLA-4, Cytotoxic T-lymphocyte associated protein 4; LRBA, Lipopolysaccharide responsive beige anchor protein; CD28, Cluster of Differentiation 28; CD80, Cluster of Differentiation 80; CD86, Cluster of Differentiation 86; PIK3Cδ, Phosphatidylinositol (4,5)-bisphosphate 3-kinase δ; PIP2, Phosphatidylinositol (4,5)-bisphosphate; PIP3, Phosphatidylinositol (3,4,5)-trisphosphate; Akt, ‘Ak’ strain ‘thymoma’ protein; mTOR, mammalian target of rapamycin; PTEN, PI3K regulatory subunit α; TCR, T cell receptor; MHCII, major histocompatibility complex Class II; NFκB1, Nuclear factor kappa B1; NFκB2, Nuclear factor kappa B2; BCR, B cell Receptor; BAFF, B cell activating factor; BAFF-R, B cell activating factor receptor; APRIL, A proliferation- inducing ligand; PLCγ2, Phospholipase C gamma 2; TACI, Transmembrane activator and calcium modulator and cyclophilin ligand interactor; CD19, Cluster of differentiation 19; CD81, Cluster of differentiation 81; TLR, Toll like receptor; Igα, Immunoglobulin alpha; Igβ, Immunoglobulin beta.

IKAROS a transcription factor in humans encoded by *IKZF1* gene. The somatic mutation in *IKZF1* gene predispose to development of malignancy while more recently patients with germline mutations have been reported to develop immunodeficiency that commonly presents as CVID. Patients with CVID with germ line mutations in *IKZF1* also develop autoimmune cytopenia. IKAROS as a transcription factor controls development of autoimmunity by promoting the B cell anergy and by regulating the TLR pathway signalling (85). It has also been shown that dimerization defective mutations in *IKZF1* gene are more likely to develop autoimmune manifestations as compared to patients with haploinsufficiency mutations. Patients with dominant negative mutations do not develop autoimmunity. The likely mechanism for an increased risk of autoimmune manifestations associated with dimerization defective mutations is an abnormal posttranslational modification of IKAROS and abnormal B cell tolerance (86). **Figure 2** illustrates various genes and downstream pathways involved in pathogenesis of autoimmune cytopenia..

Apart from above mentioned monogenic defects, few more genetic aetiologies have been identified in patients with CVID that may play an important role in the pathogenesis of immune cytopenia in these patients. The field of genetics in patients with CVID is expanding and more than 65 monogenic defects have been identified so far. It is possible that several novel genetic pathways in the pathogenesis of autoimmune cytopenia in patients with CVID would be explored as genetic aetiology of CVID is studied from other populations.

## Are Monogenic Defects More Likely to be Identified in Patients With CVID With Autoimmune Cytopenia?

Monogenic defects may account for up to 50% of all patients depending on the population studied and the techniques used. However, monogenic defects in patients with CVID have only been evaluated in few populations. It has been suggested that patients with CVID from consanguineous families, those who have an affected family member and those with unusual and refractory disease are more likely to have an underlying monogenic defect. However, because of the fact that many of these monogenic defects have an important pathophysiological link with development of autoimmune cytopenia (as discussed above), monogenic defects may possibly be identified more commonly in this subset of patients with CVID. In 2 studies that have reported monogenic defects in children with Evans syndrome (autoimmune haemolytic anaemia and thrombocytopenia), more than 2/3^rd^ patients were found to have pathogenic variants in various genes especially in the genes that also predispose to CVID such as LRBA and CTLA-4 (77, 78).

A retrospective study by Ma et al. utilised high-throughput next-generation sequencing (NGS) to identify pathogenic variants in their cohort of children with refractory ITP and it was observed that 9.1% children had pathogenic variants related to CVID {5 *TNFRSF13*; 1 *LRBA*; 1 *NF-*κ*B2*; and 1 caspase recruitment domain 11 (*CARD11*)}. Authors concluded that patients who had recurrent and/or refractory autoimmune cytopenia; propensity to develop recurrent infections and family history of autoimmunity/immunodeficiency need evaluation for underlying monogenic defects (87).

## Diagnosis and Management of Autoimmune Cytopenia in CVID

As alluded to previously, autoimmune cytopenia may be the first and only symptom of CVID. This may lead to a diagnostic conundrum as patients with CVID may not produce an adequate autoantibody response and some of the diagnostic laboratory investigations such as direct Coombs’ test, anti-platelet antibodies or anti-neutrophil antibodies may give normal results. Thus, it might be prudent to check serum immunoglobulin levels in all patients with unexplained cytopenia (5).

Glucocorticoids have remained the standard of care in autoimmune cytopenia in CVID (88). A review by Cunningham-Rundles reported that most cases of ITP/AIHA respond to oral or intravenous corticosteroids (88). Slow tapering of corticosteroids and immunoglobulin replacement is recommended. Studies have shown recurrence of cytopenia on immunoglobulin replacement therapy, however, the overall frequency as well as morbidity and mortality remain low (3, 88).

In a retrospective multicentre study on 33 patients with CVID-associated refractory immune cytopenias, rituximab showed an initial response rate of 80% and a sustained response rate of 50% at a mean follow-up of 39 months (89).

There are conflicting reports on role of splenectomy in the management of cytopenia in CVID. Initial reports showed an increased rate of mortality following splenectomy (9). However, recent studies have shown if adequate immunoglobulin replacement is being continued, splenectomy has no association with adverse outcomes (**Table 1**). Wong et al. in 2013 reported the outcome of splenectomy in patients with CVID. Splenectomy was found to be an effective long-term treatment in 75% patients with CVID with autoimmune cytopenia, even in those who were non-responsive to rituximab. Splenectomy did not increase the risk of mortality and appropriate replacement immunoglobulin therapy appeared to be sufficient for prevention of overwhelming post-splenectomy infections (90).

Current guidelines on chronic ITP and aplastic anaemia recommend the usage of thrombopoietin-receptor agonists (TPO-A) as a second or third-line agent in refractory cases (91). In CVID associated ITP, authors have suggested use of TPO-A as an alternative to splenectomy and rituximab in refractory cases (92).

Monogenic forms of CVID are often associated with autoimmune cytopenia (6). Although conventional immunosuppression and immunomodulation such as corticosteroids may work in the presence of monogenic defects, targeted therapies are now being used in these disorders (93, 94).

The mTOR pathway has been reported to be activated inn patients with APDS and mTOR inhibitor, sirolimus has been recommended for management of cytopenia and lymphoproliferation (93). However, Maccari et al. reported that sirolimus was not as effective in the management of cytopenia as it is for lymphoproliferation in these patients (95). Recently, selective PI3Kδ inhibitors such as leniolisib have also been tried (93).

Sirolimus has also been used in management of cytopenia in other monogenic forms of CVID such as LRBA deficiency and CTLA-4 haploinsufficiency with variable results. Abatacept, a CTLA-4 immunoglobulin fusion drug has been found to be an effective treatment modality for autoimmune cytopenia in these disorders. A long-term outcome study by Tesch et al. reported that disease activity scores were significantly lower in patients who were on abatacept therapy as compared to other forms of therapies (94). A recent large study on patients with CTLA-4 haploinsufficiency, cytopenia was managed using corticosteroids, rituximab, abatacept, splenectomy and immunomodulatory doses of IVIg (96). Sirolimus was not used for management of cytopenia. Following corticosteroids (that showed transient response in most patients), rituximab was the most commonly used drug and showed good efficacy in the management of cytopenia. Splenectomy produced a sustained response in 1/4^th^ of cases where it was carried out. It was also suggested that immunoglobulin replacement therapy does not prevent or ameliorate disease related complications in patients with CTLA-4 haploinsufficiency. Hematopoietic stem cell transplantation (HSCT) is an effective option for refractory cytopenia. Autoimmune cytopenia in patients with LRBA deficiency have also been managed on similar lines as in patients with CTLA-4 haploinsufficiency. However, rituximab has been used less commonly while abatacept has been used more commonly in the former group (97).

With identification of more genetic defects in patients with CVID in future, more targeted therapies are likely to be explored for management of various disease related complications such as autoimmune cytopenia.

## Conclusions

Autoimmune cytopenia is the most common autoimmune manifestation in patients with CVID. Patients with CVID with autoimmune cytopenia have unique immunophenotypic abnormalities in the B and T cell compartment. The pathophysiology of autoimmune cytopenia in CVID has been linked to an abnormality in both B and T cell compartment as well in the innate arm of the immune system. Patients with CVID with autoimmune cytopenia (especially in patients who have Evan’s syndrome) are more likely to have an underlying monogenic defect. The treatment of choice for autoimmune cytopenia in CVID remains corticosteroids. However, biologic drugs and several targeted treatments are now being explored.

## Author Contributions

SC- Preparation of the first draft of the manuscript; editing of manuscript; literature review. PB- Preparation of the first draft of the manuscript; editing of manuscript; literature review. RT-Preparation of the first draft of the manuscript; editing of manuscript; literature review. AJ- Preparation of the first draft of the manuscript; editing of manuscript; literature review; critical review and final approval. SSh/AR- Editing of manuscript, review of literature. SSi- Critical revision of manuscript, review of literature and final approval. All authors contributed to the article and approved the submitted version.

## Conflict of Interest

The authors declare that the research was conducted in the absence of any commercial or financial relationships that could be construed as a potential conflict of interest.

## Publisher’s Note

All claims expressed in this article are solely those of the authors and do not necessarily represent those of their affiliated organizations, or those of the publisher, the editors and the reviewers. Any product that may be evaluated in this article, or claim that may be made by its manufacturer, is not guaranteed or endorsed by the publisher.

## References

[1] Cunningham-Rundles C. Hematologic Complications of Primary Immune Deficiencies. Blood Rev (2002) 16(1):61–4. doi: 10.1054/blre.2001.0185 11913998

[2] Bonilla FA Barlan I Chapel H Costa-Carvalho BT Cunningham-Rundles C de la Morena MT. International Consensus Document (ICON): Common Variable Immunodeficiency Disorders. J Allergy Clin Immunol Pract (2016) 4(1):38–59. doi: 10.1016/j.jaip.2015.07.025 26563668 PMC4869529

[3] Azizi G Abolhassani H Asgardoon MH Alinia T Yazdani R Mohammadi J. Autoimmunity in Common Variable Immunodeficiency: Epidemiology, Pathophysiology and Management. Expert Rev Clin Immunol (2017) 13(2):101–15. doi: 10.1080/1744666X.2016.1224664 27636680

[4] Sanford JP Favour CB Tribeman MS. Absence of Serum Gamma Globulins in an Adult. N Engl J Med (1954) 250(24):1027–9. doi: 10.1056/NEJM195406172502403 13165949

[5] Podjasek JC Abraham RS. Autoimmune Cytopenias In Common Variable Immunodeficiency. Front Immunol (2012) 38:28. doi: 10.3389/fimmu.2012.00189 PMC3402902 22837758

[6] Bogaert DJA Dullaers M Lambrecht BN Vermaelen KY De Baere E Haerynck F. Genes Associated With Common Variable Immunodeficiency: One Diagnosis to Rule Them All? J Med Genet (2016) 53(9):575–90. doi: 10.1136/jmedgenet-2015-103690 27250108

[7] Asgardoon MH Azizi G Yazdani R Sohani M Pashangzadeh S Kalantari A. Monogenic Primary Immunodeficiency Disorder Associated With Common Variable Immunodeficiency and Autoimmunity. Int Arch Allergy Immunol (2020) 181(9):706–14. doi: 10.1159/000508817 32615565

[8] Feuille EJ Anooshiravani N Sullivan KE Fuleihan RL Cunningham-Rundles C. Autoimmune Cytopenias and Associated Conditions in CVID: A Report From the USIDNET Registry. J Clin Immunol (2018) 38(1):28–34. doi: 10.1007/s10875-017-0456-9 29080979 PMC5743637

[9] Gathmann B Mahlaoui N Gérard L Oksenhendler E Warnatz K Schulze I. Clinical Picture and Treatment of 2212 Patients With Common Variable Immunodeficiency. J Allergy Clin Immunol (2014) 134(1):116–26.e11. doi: 10.1016/j.jaci.2013.12.1077 24582312

[10] Mormile I Punziano A Riolo CA Granata F Williams M de Paulis A. Common Variable Immunodeficiency and Autoimmune Diseases: A Retrospective Study of 95 Adult Patients in a Single Tertiary Care Center. Front Immunol (2021) 12:652487. doi: 10.3389/fimmu.2021.652487 34290696 PMC8287325

[11] Hermaszewski RA Webster AD. Primary Hypogammaglobulinaemia: A Survey of Clinical Manifestations and Complications. Q J Med (1993) 86(1):31–42. 8438047

[12] Cunningham-Rundles C Bodian C. Common Variable Immunodeficiency: Clinical and Immunological Features of 248 Patients. Clin Immunol (1999) 92(1):34–48. doi: 10.1006/clim.1999.4725 10413651

[13] Kainulainen L Nikoskelainen J Ruuskanen O. Diagnostic Findings in 95 Finnish Patients With Common Variable Immunodeficiency. J Clin Immunol (2001) 21(2):145–9. doi: 10.1023/A:1011012023616 11332653

[14] Kokron CM Errante PR Barros MT Baracho GV Camargo MM Kalil J. Clinical and Laboratory Aspects of Common Variable Immunodeficiency. Acad Bras Ciênc (2004) 76(4):707–26. doi: 10.1590/S0001-37652004000400007 15558152

[15] Michel M Chanet V Galicier L Ruivard M Levy Y Hermine O. Autoimmune Thrombocytopenic Purpura and Common Variable Immunodeficiency: Analysis of 21 Cases and Review of the Literature. Med (Baltimore) (2004) 83(4):254–63. doi: 10.1097/01.md.0000133624.65946.40 15232313

[16] Wang J Cunningham-Rundles C. Treatment and Outcome of Autoimmune Hematologic Disease in Common Variable Immunodeficiency (CVID). J Autoimmun (2005) 25(1):57–62. doi: 10.1016/j.jaut.2005.04.006 15994061

[17] Carbone J Sarmiento E Micheloud D Rodríguez-Molina J Fernández-Cruz E. Elevated Levels of Activated CD4 T Cells in Common Variable Immunodeficiency: Association With Clinical Findings. Allergol Immunopathol (Madr) (2006) 34(4):131–5. doi: 10.1157/13091037 16854344

[18] Alachkar H Taubenheim N Haeney MR Durandy A Arkwright PD. Memory Switched B Cell Percentage and Not Serum Immunoglobulin Concentration is Associated With Clinical Complications in Children and Adults With Specific Antibody Deficiency and Common Variable Immunodeficiency. Clin Immunol (2006) 120(3):310–8. doi: 10.1016/j.clim.2006.05.003 16782407

[19] Quinti I Soresina A Spadaro G Martino S Donnanno S Agostini C. Long-Term Follow-Up and Outcome of a Large Cohort of Patients With Common Variable Immunodeficiency. J Clin Immunol (2007) 27(3):308–16. doi: 10.1007/s10875-007-9075-1 17510807

[20] Chapel H Lucas M Lee M Bjorkander J Webster D Grimbacher B. Common Variable Immunodeficiency Disorders: Division Into Distinct Clinical Phenotypes. Blood (2008) 112(2):277–86. doi: 10.1182/blood-2007-11-124545 18319398

[21] Wehr C Kivioja T Schmitt C Ferry B Witte T Eren E. The EUROclass Trial: Defining Subgroups in Common Variable Immunodeficiency. Blood (2008) 111(1):77–85. doi: 10.1182/blood-2007-06-091744 17898316

[22] Ardeniz Ö Cunningham-Rundles C. Granulomatous Disease in Common Variable Immunodeficiency. Clin Immunol (2009) 133(2):198–207. doi: 10.1016/j.clim.2009.05.001 19716342 PMC2760682

[23] for the DEFI Study Group Mouillot G Carmagnat M Gérard L Garnier J-L Fieschi C. B-Cell and T-Cell Phenotypes in CVID Patients Correlate With the Clinical Phenotype of the Disease. J Clin Immunol (2010) 30(5):746–55. doi: 10.1007/s10875-010-9424-3 20437084

[24] Boileau J Mouillot G Gérard L Carmagnat M Rabian C Oksenhendler E. Autoimmunity in Common Variable Immunodeficiency: Correlation With Lymphocyte Phenotype in the French DEFI Study. J Autoimmun (2011) 36(1):25–32. doi: 10.1016/j.jaut.2010.10.002 21075598

[25] Maarschalk-Ellerbroek LJ Hoepelman AIM van Montfrans JM Ellerbroek PM. The Spectrum of Disease Manifestations in Patients With Common Variable Immunodeficiency Disorders and Partial Antibody Deficiency in a University Hospital. J Clin Immunol (2012) 32(5):907–21. doi: 10.1007/s10875-012-9671-6 PMC3443482 22526591

[26] Arshi S Nabavi M Bemanian MH Shakeri R Taghvaei B Ghalebaghi B. Phenotyping and Follow Up of Forty-Seven Iranian Patients With Common Variable Immunodeficiency. Allergol Immunopathol (Madr) (2016) 44(3):226–31. doi: 10.1016/j.aller.2015.04.005 26232306

[27] Patuzzo G Barbieri A Tinazzi E Veneri D Argentino G Moretta F. Autoimmunity and Infection in Common Variable Immunodeficiency (CVID). Autoimmun Rev (2016) 15(9):877–82. doi: 10.1016/j.autrev.2016.07.011 27392505

[28] Arduini S Dunne J Conlon N Feighery C Doherty DG. Mucosal-Associated Invariant T Cells are Depleted and Functionally Altered in Patients With Common Variable Immunodeficiency. Clin Immunol (2017) 176:23–30. doi: 10.1016/j.clim.2016.12.002 28011187

[29] Çalişkaner AZ Reisli İ Arslan Ş Uçar R Ataseven H Selçuk NY. Common Variable Immunodeficiency in Adults Requires Reserved Protocols for Long-Term Follow-Up. Turk J Med Sci (2016) 46(2):430–6. doi: 10.3906/sag-1412-108 27511507

[30] Almejún MB Campos BC Patiño V Galicchio M Zelazko M Oleastro M. Noninfectious Complications in Patients With Pediatric-Onset Common Variable Immunodeficiency Correlated With Defects in Somatic Hypermutation But Not in Class-Switch Recombination. J Allergy Clin Immunol (2017) 139(3):913–22. doi: 10.1016/j.jaci.2016.08.030 27713077

[31] the DEFI study group Guffroy A Mourot-Cottet R Gérard L Gies V Lagresle C. Neutropenia in Patients With Common Variable Immunodeficiency: A Rare Event Associated With Severe Outcome. J Clin Immunol (2017) 37(7):715–26. doi: 10.1007/s10875-017-0434-2 28842786

[32] Alkan G Keles S Reisli İ. Evaluation of Clinical and Immunological Characteristics of Children With Common Variable Immunodeficiency. Int J Pediatr (2018) 2018:1–8. doi: 10.1155/2018/3527480 PMC5937368 29849668

[33] Ghorbani M Fekrvand S Shahkarami S Yazdani R Sohani M Shaghaghi M. The Evaluation of Neutropenia in Common Variable Immune Deficiency Patients. Expert Rev Clin Immunol (2019) 15(11):1225–33. doi: 10.1080/1744666X.2020.1677154 31592698

[34] Rizvi FS Zainaldain H Rafiemanesh H Jamee M Hossein-Khannazer N Hamedifar H. Autoimmunity in Common Variable Immunodeficiency: A Systematic Review and Meta-Analysis. Expert Rev Clin Immunol (2020) 16(12):1227–35. doi: 10.1080/1744666X.2021.1850272 33203275

[35] Conn HO. Pernicious Anemia and Immunologic Deficiency. Ann Intern Med (1968) 68(3):603. doi: 10.7326/0003-4819-68-3-603 4171211

[36] Bizzaro N Antico A. Diagnosis and Classification of Pernicious Anemia. Autoimmun Rev (2014) 13(4-5):565–8. doi: 10.1016/j.autrev.2014.01.042 24424200

[37] Kalha I Sellin JH. Common Variable Immunodeficiency and the Gastrointestinal Tract. Curr Gastroenterol Rep (2004) 6(5):377–83. doi: 10.1007/s11894-004-0053-y 15341713

[38] Moriuchi H Takayanagi T Yamasaki S Yasui M Mori K Yanai M. Pernicious Anemia in a Patient With Hypogammaglobulinemia. Pediatr Int (1990) 32(3):311–4. doi: 10.1111/j.1442-200X.1990.tb00830.x 2239305

[39] Warnatz K Denz A Dräger R Braun M Groth C Wolff-Vorbeck G. Severe Deficiency of Switched Memory B Cells (CD27(+)IgM(-)IgD(-)) in Subgroups of Patients With Common Variable Immunodeficiency: A New Approach to Classify a Heterogeneous Disease. Blood (2002) 99(5):1544–51. doi: 10.1182/blood.V99.5.1544 11861266

[40] Kofod-Olsen E Jørgensen SE Nissen SK Westh L Møller BK Østergaard L. Altered Fraction of Regulatory B and T Cells is Correlated With Autoimmune Phenomena and Splenomegaly in Patients With CVID. Clin Immunol (2016) 162:49–57. doi: 10.1016/j.clim.2015.11.003 26586095

[41] Genre J Errante PR Kokron CM Toledo-Barros M Câmara NOS Rizzo LV. Reduced Frequency of CD4+CD25HIGHFOXP3+ Cells and Diminished FOXP3 Expression in Patients With Common Variable Immunodeficiency: A Link to Autoimmunity? Clin Immunol (2009) 132(2):215–21. doi: 10.1016/j.clim.2009.03.519 19394278

[42] Tahiat A Djidjik R Boushaki S Cherguelaïne K Gharnaout M Boumedine S. Common Variable Immunodeficiency (CVID): Clinical and Immunological Features of 29 Algerian Patients. Pathol Biol (Paris) (2014) 62(6):377–81. doi: 10.1016/j.patbio.2014.04.002 25200463

[43] Romberg N Le Coz C Glauzy S Schickel J-N Trofa M Nolan BE. CVID Patients With Autoimmune Cytopenias Exhibit Hyperplastic Yet Inefficient Germinal Center Responses. J Allergy Clin Immunol (2019) 143(1):258–65. doi: 10.1016/j.jaci.2018.06.012 PMC6400323 29935219

[44] Agarwal S Cunningham-Rundles C. Autoimmunity in Common Variable Immunodeficiency. Curr Allergy Asthma Rep (2009) 9(5):347–52. doi: 10.1007/s11882-009-0051-0 PMC2919211 19671377

[45] Lopez-Herrera G Tampella G Pan-Hammarström Q Herholz P Trujillo-Vargas CM Phadwal K. Deleterious Mutations in LRBA are Associated With a Syndrome of Immune Deficiency and Autoimmunity. Am J Hum Genet (2012) 90(6):986–1001. doi: 10.1016/j.ajhg.2012.04.015 22608502 PMC3370280

[46] Cashman KS Jenks SA Woodruff MC Tomar D Tipton CM Scharer CD. Understanding and Measuring Human B Cell Tolerance and its Breakdown in Autoimmune Disease. Immunol Rev (2019) 292(1):76–89. doi: 10.1111/imr.12820 31755562 PMC6935423

[47] Isnardi I Ng Y-S Menard L Meyers G Saadoun D Srdanovic I. Complement Receptor 2/CD21– Human Naive B Cells Contain Mostly Autoreactive Unresponsive Clones. Blood (2010) 115(24):5026–36. doi: 10.1182/blood-2009-09-243071 PMC3373152 20231422

[48] Lesley R Xu Y Kalled SL Hess DM Schwab SR Shu H-B. Reduced Competitiveness of Autoantigen-Engaged B Cells Due to Increased Dependence on BAFF. Immunity (2004) 20(4):441–53. doi: 10.1016/S1074-7613(04)00079-2 15084273

[49] Lyubchenko T Dal Porto JM Holers VM Cambier JC. Cutting Edge: Complement (C3d)-Linked Antigens Break B Cell Anergy. J Immunol (2007) 179(5):2695–9. doi: 10.4049/jimmunol.179.5.2695 17709481

[50] Yu GP Chiang D Song SJ Hoyte EG Huang J Vanishsarn C. Regulatory T Cell Dysfunction in Subjects With Common Variable Immunodeficiency Complicated by Autoimmune Disease. Clin Immunol Orlando Fla. (2009) 131(2):240–53. doi: 10.1016/j.clim.2008.12.006 PMC5140037 19162554

[51] Bateman EAL Ayers L Sadler R Lucas M Roberts C Woods A. T Cell Phenotypes in Patients With Common Variable Immunodeficiency Disorders: Associations With Clinical Phenotypes in Comparison With Other Groups With Recurrent Infections: T Cell Phenotypes in CVID and Other PADs. Clin Exp Immunol (2012) 170(2):202–11. doi: 10.1111/j.1365-2249.2012.04643.x PMC3482367 23039891

[52] Piqueras B Lavenu-Bombled C Galicier L Bergeron-van der Cruyssen F Mouthon L Chevret S. Common Variable Immunodeficiency Patient Classification Based on Impaired B Cell Memory Differentiation Correlates With Clinical Aspects. J Clin Immunol (2003) 23(5):385–400. doi: 10.1023/A:1025373601374 14601647

[53] Horn J Manguiat A Berglund LJ Knerr V Tahami F Grimbacher B. Decrease in Phenotypic Regulatory T Cells in Subsets of Patients With Common Variable Immunodeficiency. Clin Exp Immunol (2009) 156(3):446–54. doi: 10.1111/j.1365-2249.2009.03913.x PMC2691973 19438597

[54] Taraldsrud E Fevang B Aukrust P Beiske KH Fløisand Y Frøland S. Common Variable Immunodeficiency Revisited: Normal Generation of Naturally Occurring Dendritic Cells That Respond to Toll-Like Receptors 7 and 9. Clin Exp Immunol (2014) 175(3):439–48. doi: 10.1111/cei.12239 PMC3927904 24237110

[55] Sharifi L Mirshafiey A Rezaei N Azizi G Magaji Hamid K Amirzargar AA. The Role of Toll-Like Receptors in B-Cell Development and Immunopathogenesis of Common Variable Immunodeficiency. Expert Rev Clin Immunol (2016) 12(2):195–207. doi: 10.1586/1744666X.2016.1114885 26654573

[56] Rezaei N Amirzargar AA Shakiba Y Mahmoudi M Moradi B Aghamohammadi A. Proinflammatory Cytokine Gene Single Nucleotide Polymorphisms in Common Variable Immunodeficiency. Clin Exp Immunol (2008) 155(1):21–7. doi: 10.1111/j.1365-2249.2008.03790.x PMC2665675 19076825

[57] Azizi G Abolhassani H Kiaee F Tavakolinia N Rafiemanesh H Yazdani R. Autoimmunity and its Association With Regulatory T Cells and B Cell Subsets in Patients With Common Variable Immunodeficiency. Allergol Immunopathol (Madr) (2018) 46(2):127–35. doi: 10.1016/j.aller.2017.04.004 28735808

[58] Barcellini W Zaninoni A Giannotta JA Fattizzo B. New Insights in Autoimmune Hemolytic Anemia: From Pathogenesis to Therapy Stage 1. J Clin Med (2020) 9(12):3859. doi: 10.3390/jcm9123859 PMC7759854 33261023

[59] Galanopoulos N Christoforidou A Bezirgiannidou Z. Lupus Thrombocytopenia:Pathogenesis and Therapeutic Implications. Mediterr J Rheumatol (2017) 28(1):20–6. doi: 10.31138/mjr.28.1.20 PMC7045921 32185250

[60] Shah S Wu E Rao VK Tarrant TK. Autoimmune Lymphoproliferative Syndrome: An Update and Review of the Literature. Curr Allergy Asthma Rep (2014) 14(9):462. doi: 10.1007/s11882-014-0462-4 25086580 PMC4148697

[61] Kakkas I Tsinti G Kalala F Farmaki E Kourakli A Kapousouzi A. TACI Mutations in Primary Antibody Deficiencies: A Nationwide Study in Greece. Medicina (Mex) (2021) 57(8):827. doi: 10.3390/medicina57080827 PMC8401742 34441032

[62] Romberg N Chamberlain N Saadoun D Gentile M Kinnunen T Ng YS. CVID-Associated TACI Mutations Affect Autoreactive B Cell Selection and Activation. J Clin Invest (2013) 123(10):4283–93. doi: 10.1172/JCI69854 PMC3786721 24051380

[63] Salzer U Grimbacher B. TACI Deficiency — a Complex System Out of Balance. Curr Opin Immunol (2021) 71:81–8. doi: 10.1016/j.coi.2021.06.004 34247095

[64] Gereige JD Maglione PJ. Current Understanding and Recent Developments in Common Variable Immunodeficiency Associated Autoimmunity. Front Immunol (2019) 10:2753. doi: 10.3389/fimmu.2019.02753 31921101 PMC6914703

[65] Schneider P MacKay F Steiner V Hofmann K Bodmer JL Holler N. BAFF, a Novel Ligand of the Tumor Necrosis Factor Family, Stimulates B Cell Growth. J Exp Med (1999) 189(11):1747–56. doi: 10.1084/jem.189.11.1747 PMC2193079 10359578

[66] Moore PA Belvedere O Orr A Pieri K LaFleur DW Feng P. BLyS: Member of the Tumor Necrosis Factor Family and B Lymphocyte Stimulator. Science (1999) 285(5425):260–3. doi: 10.1126/science.285.5425.260 10398604

[67] Stohl W Metyas S Tan S-M Cheema GS Oamar B Xu D. B Lymphocyte Stimulator Overexpression in Patients With Systemic Lupus Erythematosus: Longitudinal Observations. Arthritis Rheumatol (2003) 48(12):3475–86. doi: 10.1002/art.11354 14673998

[68] Seyler TM Park YW Takemura S Bram RJ Kurtin PJ Goronzy JJ. BLyS and APRIL in Rheumatoid Arthritis. J Clin Invest (2005) 115(11):3083–92. doi: 10.1172/JCI25265 PMC1257539 16239971

[69] Szodoray P Jonsson R. The BAFF/APRIL System in Systemic Autoimmune Diseases With a Special Emphasis on Sjögren’s Syndrome. Scand J Immunol (2005) 62(5):421–8. doi: 10.1111/j.1365-3083.2005.01688.x 16305638

[70] Zhu X Shi Y Peng J Guo C Shan N Qin P. The Effects of BAFF and BAFF-R-Fc Fusion Protein in Immune Thrombocytopenia. Blood (2009) 114(26):5362–7. doi: 10.1182/blood-2009-05-217513 19794139

[71] Liu Z Davidson A. BAFF and Selection of Autoreactive B Cells. Trends Immunol (2011) 32(8):388–94. doi: 10.1016/j.it.2011.06.004 PMC3151317 21752714

[72] Abolhassani H El-Sherbiny YM Arumugakani G Carter C Richards S Lawless D. Expanding Clinical Phenotype and Novel Insights Into the Pathogenesis of ICOS Deficiency. J Clin Immunol (2020) 40(2):277–88. doi: 10.1007/s10875-019-00735-z PMC7082411 31858365

[73] Warnatz K. Human ICOS Deficiency Abrogates the Germinal Center Reaction and Provides a Monogenic Model for Common Variable Immunodeficiency. Blood (2006) 107(8):3045–52. doi: 10.1182/blood-2005-07-2955 16384931

[74] Schepp J Chou J Skrabl-Baumgartner A Arkwright PD Engelhardt KR Hambleton S. 14 Years After Discovery: Clinical Follow-Up on 15 Patients With Inducible Co-Stimulator Deficiency. Front Immunol (2017) 8:964. doi: 10.3389/fimmu.2017.00964 28861081 PMC5561331

[75] Gámez-Díaz L Grimbacher B. Immune Checkpoint Deficiencies and Autoimmune Lymphoproliferative Syndromes. BioMed J (2021) 44(4):400–11. doi: 10.1016/j.bj.2021.04.005 PMC8514790 34384744

[76] Abolhassani H Hammarström L Cunningham-Rundles C. Current Genetic Landscape in Common Variable Immune Deficiency. Blood (2020) 135(9):656–67. doi: 10.1182/blood.2019000929 PMC7046605 31942606

[77] Hadjadj J Aladjidi N Fernandes H Leverger G Magérus-Chatinet A Mazerolles F. Pediatric Evans Syndrome is Associated With a High Frequency of Potentially Damaging Variants in Immune Genes. Blood (2019) 134(1):9–21. doi: 10.1182/blood-2018-11-887141 30940614

[78] Besnard C Levy E Aladjidi N Stolzenberg M-C Magerus-Chatinet A Alibeu O. Pediatric-Onset Evans Syndrome: Heterogeneous Presentation and High Frequency of Monogenic Disorders Including LRBA and CTLA4 Mutations. Clin Immunol (2018) 188:52–7. doi: 10.1016/j.clim.2017.12.009 29330115

[79] van Zelm MC Smet J Adams B Mascart F Schandené L Janssen F. CD81 Gene Defect in Humans Disrupts CD19 Complex Formation and Leads to Antibody Deficiency. J Clin Invest (2010) 120(4):1265–74. doi: 10.1172/JCI39748 PMC2846042 20237408

[80] Jamee M Moniri S Zaki-Dizaji M Olbrich P Yazdani R Jadidi-Niaragh F. Clinical, Immunological, and Genetic Features in Patients With Activated Pi3kδ Syndrome (APDS): A Systematic Review. Clin Rev Allergy Immunol (2020) 59(3):323–33. doi: 10.1007/s12016-019-08738-9 31111319

[81] Schworer SA Francis OL Johnson SM Smith BD Gold SH Smitherman AB. Autoimmune Cytopenia as an Early and Initial Presenting Manifestation in Activated PI3 Kinase Delta Syndrome: Case Report and Review. J Pediatr Hematol Oncol (2021) 43(8):281–7. doi: 10.1097/MPH.0000000000002214 PMC8542580 34054047

[82] Singh A Joshi V Jindal AK Mathew B Rawat A. An Updated Review on Activated PI3 Kinase Delta Syndrome (APDS). Genes Dis (2020) 7(1):67–74. doi: 10.1016/j.gendis.2019.09.015 32181277 PMC7063426

[83] Preite S Gomez-Rodriguez J Cannons JL Schwartzberg PL. T and B-Cell Signaling in Activated PI3K Delta Syndrome: From Immunodeficiency to Autoimmunity. Immunol Rev (2019) 291(1):154–73. doi: 10.1111/imr.12790 31402502

[84] Klemann C Camacho-Ordonez N Yang L Eskandarian Z Rojas-Restrepo JL Frede N. Clinical and Immunological Phenotype of Patients With Primary Immunodeficiency Due to Damaging Mutations in NFKB2. Front Immunol (2019) 10:297. doi: 10.3389/fimmu.2019.00297 30941118 PMC6435015

[85] Schwickert TA Tagoh H Schindler K Fischer M Jaritz M Busslinger M. Ikaros Prevents Autoimmunity by Controlling Anergy and Toll-Like Receptor Signaling in B Cells. Nat Immunol (2019) 20(11):1517–29. doi: 10.1038/s41590-019-0490-2 PMC7115902 31591571

[86] Kuehn HS Nunes-Santos CJ Rosenzweig SD. Germline *IKZF1* Mutations and Their Impact on Immunity: IKAROS-Associated Diseases and Pathophysiology. Expert Rev Clin Immunol (2021) 17(4):407–16. doi: 10.1080/1744666X.2021.1901582 PMC8091572 33691560

[87] Ma J Fu L Gu H Chen Z Zhang J Zhao S. Screening for Genetic Mutations for the Early Diagnosis of Common Variable Immunodeficiency in Children With Refractory Immune Thrombocytopenia: A Retrospective Data Analysis From a Tertiary Children's Center. Front Pediatr (2020) 8:595135. doi: 10.3389/fped.2020.595135 33425813 PMC7793988

[88] Cunningham-Rundles C. Common Variable Immune Deficiency: Case Studies. Hematology (2019) 2019(1):449–56. doi: 10.1182/hematology.2019002062 PMC6913496 31808912

[89] Gobert D Bussel JB Cunningham-Rundles C Galicier L Dechartres A Berezne A. Efficacy and Safety of Rituximab in Common Variable Immunodeficiency-Associated Immune Cytopenias: A Retrospective Multicentre Study on 33 Patients: Efficacy and Safety of Rituximab. Br J Haematol (2011) 155(4):498–508. doi: 10.1111/j.1365-2141.2011.08880.x 21981575 PMC3428031

[90] Wong GK Goldacker S Winterhalter C Grimbacher B Chapel H Lucas M. Outcomes of Splenectomy in Patients With Common Variable Immunodeficiency (CVID): A Survey of 45 Patients. Clin Exp Immunol (2013) 172(1):63–72. doi: 10.1111/cei.12039 23480186 PMC3719932

[91] Provan D Arnold DM Bussel JB Chong BH Cooper N Gernsheimer T. Updated International Consensus Report on the Investigation and Management of Primary Immune Thrombocytopenia. Blood Adv (2019) 3(22):3780–817. doi: 10.1182/bloodadvances.2019000812 PMC6880896 31770441

[92] Carrabba M Barcellini W Fabio G. Use of Thrombopoietin-Receptor Agonist in CVID-Associated Immune Thrombocytopenia. J Clin Immunol (2016) 36(5):434–6. doi: 10.1007/s10875-016-0282-5 27072856

[93] Coulter TI Cant AJ. The Treatment of Activated Pi3kδ Syndrome. Front Immunol (2018) 9:2043. doi: 10.3389/fimmu.2018.02043 30245694 PMC6137162

[94] Tesch VK Abolhassani H Shadur B Zobel J Mareika Y Sharapova S. Long-Term Outcome of LRBA Deficiency in 76 Patients After Various Treatment Modalities as Evaluated by the Immune Deficiency and Dysregulation Activity (IDDA) Score. J Allergy Clin Immunol (2020) 145(5):1452–63. doi: 10.1016/j.jaci.2019.12.896 31887391

[95] Maccari ME Abolhassani H Aghamohammadi A Aiuti A Aleinikova O Bangs C. Disease Evolution and Response to Rapamycin in Activated Phosphoinositide 3-Kinase δ Syndrome: The European Society for Immunodeficiencies-Activated Phosphoinositide 3-Kinase δ Syndrome Registry. Front Immunol (2018) 9:543. doi: 10.3389/fimmu.2018.00543 29599784 PMC5863269

[96] Egg D Rump IC Mitsuiki N Rojas-Restrepo J Maccari M-E Schwab C. Therapeutic Options for CTLA-4 Insufficiency. J Allergy Clin Immunol (2021) 149(2):736–46. doi: 10.1016/j.jaci.2021.04.039 34111452

[97] Jamee M Hosseinzadeh S Sharifinejad N Zaki-Dizaji M Matloubi M Hasani M. Comprehensive Comparison Between 222 CTLA-4 Haploinsufficiency and 212 LRBA Deficiency Patients: A Systematic Review. Clin Exp Immunol (2021) 205(1):28–43. doi: 10.1111/cei.13600 33788257 PMC8209572

